# KIF1A mediates axonal transport of BACE1 and identification of independently moving cargoes in living SCG neurons

**DOI:** 10.1111/tra.12428

**Published:** 2016-10-05

**Authors:** Christy O. Y. Hung, Michael P. Coleman

**Affiliations:** ^1^ Department of Signalling ProgrammeBabraham InstituteCambridgeUK; ^2^ John van Geest Centre for Brain RepairUniversity of CambridgeCambridgeUK

**Keywords:** axonal transport, BACE1, KIF1A, live‐cell imaging

## Abstract

Neurons rely heavily on axonal transport to deliver materials from the sites of synthesis to the axon terminals over distances that can be many centimetres long. KIF1A is the neuron‐specific kinesin with the fastest reported anterograde motor activity. Previous studies have shown that KIF1A transports a subset of synaptic proteins, neurofilaments and dense‐core vesicles. Using two‐colour live imaging, we showed that beta‐secretase 1 (BACE1)‐mCherry moves together with KIF1A‐GFP in both the anterograde and retrograde directions in superior cervical ganglions (SCG) neurons. We confirmed that KIF1A is functionally required for BACE1 transport by using KIF1A siRNA and a KIF1A mutant construct (KIF1A‐T312M) to impair its motor activity. We further identified several cargoes that have little or no co‐migration with KIF1A‐GFP and also move independently from BACE1‐mCherry. Together, these findings support a primary role for KIF1A in the anterograde transport of BACE1 and suggest that axonally transported cargoes are sorted into different classes of carrier vesicles in the cell body and are transported by cargo‐specific motor proteins through the axon.

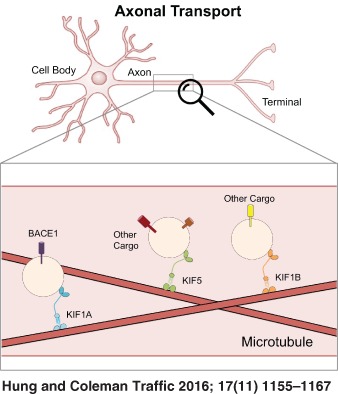

## 1 INTRODUCTION

The kinesin superfamily proteins (KIFs) are a large gene family of microtubule‐dependent motors; over 45 members have been identified in the mammalian genome.^1^ A wide array of cellular cargoes are simultaneously transported within an axon by different motor proteins, including transport vesicles, membranous organelle and non‐membranous cargoes such as cytoskeletal filaments, cytosolic protein complexes and messenger RNAs.^2^ As motor‐cargo interactions are specific, it is difficult to recognise the specific cargoes of a motor protein among the multitude of other cargoes that are present in the sheer volume of axons.^3^ As the types of cargo greatly outnumber the motors, motors must recognise and carry more than one type of cargo for efficient transport, but there appears also to be some redundancy in which motor protein carries which cargo. The identification of the cargoes that each motor carries is one of the most poorly developed areas in the field of microtubule‐dependent trafficking.^4^ Previous studies addressing this question have been largely dependent on co‐immunoprecipitation strategies. These studies have led to the identification of important binding partners, 5, 6, 7, 8 but with two major caveats. First, as stationary material will also be precipitated, this approach alone does not show that a motor actually moves the associated cargo. Second, this method may fail to pull‐down membranous cargoes that do not interact directly with the motor protein, especially if the membranes are disrupted during extraction. Transient interactions with motor protein during transport may also be missed. Alternative methods to address this question, such as co‐migration analysis in live imaging and the effect of motor protein depletion on cargo movement, have been relatively little used.

KIF1A belongs to the Kinesin‐3 family.^9^ KIF1A is a neuron‐specific kinesin and it also has the fastest reported anterograde motor activity.^10^ It is suggested to act as a monomer,^11^ although Hammond et al reported that KIF1A motors exist in a dimeric state and only dimerized motors display ATP‐dependent processive motility.^12^ Previous studies have shown that KIF1A transports a subset of synaptic proteins 1, 4 and dense‐core vesicles.^13^ However, the identity of other cargoes carried by KIF1A is still largely unknown. As a good live imaging method is already available for KIF1A,^14^ we decided to study which cargoes co‐migrate with this motor in dissociated superior cervical ganglions (SCG) neurons. SCG neurons have been used extensively to study axonal transport, largely because the polarity of cell processes (axons vs dendrites) can be easily determined in the absence of markers. This, together with their convenient genetic manipulation by microinjection,^15^ provides us with a good in vitro model for evaluating transport kinetics, such as the percentage of moving vesicles and the average and maximum velocities in both the anterograde and retrograde directions.

The deposition of amyloid β (Aβ) peptide is the major pathological hallmark of Alzheimer's disease. Beta‐site amyloid precursor protein (APP) cleaving enzyme 1 [beta‐secretase 1 (BACE1)], a transmembrane aspartyl protease, initiates the processing of the APP during Aβ generation and therefore is the rate‐limiting enzyme in the amyloidogenic pathway.^16^ As APP and BACE1 are highly expressed in neurons, it is reasonable to assume there should be a mechanism that segregates the transport of APP and BACE1 to prevent overproduction of Aβ. Despite extensive research, the problem of where wild‐type APP and BACE1 meet and the location of the β‐cleavage site remain controversial. Previous studies have suggested that APP is transported in the same axonal vesicle as BACE1 and γ‐secretase, that APP can then be cleaved by them, and consequently Aβ is produced in these vesicles.^17^ This view, however, has been challenged by several studies. A ligation experiment on sciatic nerves found that APP is not co‐transported with BACE1.^18^ This finding was further supported by a video microscopy study showing that APP is transported on a vesicle distinct from BACE1.^19^ A recent study using an optical assay, however, found that APP and BACE1 were co‐transported in axons and that they interacted during transit.^20^ Understanding axonal BACE1 transport is important because abnormal accumulation of BACE1 at the presynaptic terminals has been reported in post‐mortem AD brain.^21^ This finding suggests that elevation of local BACE1 level could promote the generation of Aβ and this could contribute to the development of AD.^22^ However, the molecular players involved in BACE1 transport in axons are still not completely identified.

In this study, we first characterized the axonal transport of a panel of cargo candidates, ranging from signalling molecules to proteins that are involved in neurodegenerative disorders. Two‐colour live imaging showed that BACE1‐mCherry moves together with KIF1A‐GFP in both the anterograde and retrograde directions. We identified several cargoes that show little or no co‐migration with KIF1A‐GFP and demonstrated that these also move independently from BACE1‐mCherry. Our findings support a primary role for KIF1A in the anterograde transport of BACE1 and suggest the existence of independently moving groups of cargoes in neurons. KIF1A is only responsible for the transport of a sub‐population of these moving cargoes.

## 2 RESULTS

### 2.1 Identification of axonally transported cargoes that can be easily tracked in SCG axons using live imaging

**Figure 1:**
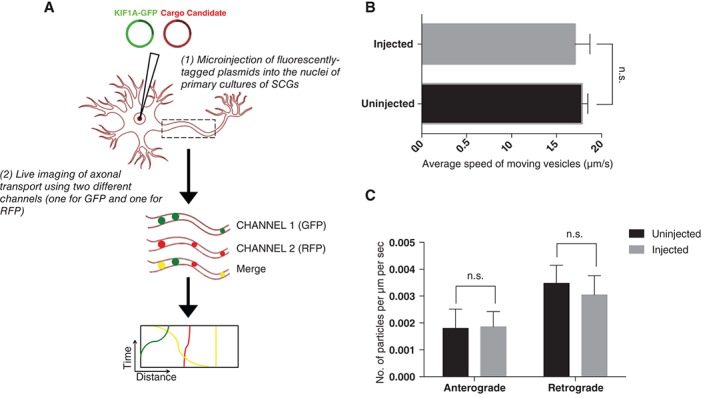
(A) Schematic diagram for axonal transport experiments. Dissociated superior cervical ganglions (SCG) neurons were microinjected with GFP‐tagged KIF1A with different cargo candidates constructs, vesicle transport in axons was imaged live after 6‐8 hours, and resultant kymographs were analyzed. (B) Average speed of moving lysosomes (µm/seconds) in cells injected with a GFP construct (n = 19 neurites) and uninjected cells (n = 11 neurites). Error bars indicate standard error of the mean (SEM). (C) Quantification of the number of moving lysosomes in injected (n = 19 neurites) cells and uninjected cells (n = 11 neurites). Error bars indicate SEM. The direction, velocity and number of moving lysosomes in both the anterograde and retrograde directions are similar in the cells that have been injected as in uninjected cells.

The aim of this study was to identify the cargoes that KIF1A carried. Therefore we first identified cargoes that can be easily tracked in SCG axons using live cell imaging. We visualized the axonal transport of a panel of cargoes chosen from different categories, ranging from signalling molecules to proteins that are involved in neurodegenerative disorders (see Table 1). We first fused each cargo to a fluorescent tag (either mCherry or RFP). We then microinjected a low level of construct encoding fluorescently labelled protein into the nuclei of 7DIV SCG dissociated cultures and visualized the transport of the fluorescently labelled protein in the axons at 37°C 6‐8 hours after microinjection (schematic in Figure 1A). Microinjected cells that appeared healthy by phase‐contrast were imaged by video microscopy. To determine whether the use of microinjection can physically damage the injected cells and thus affect vesicle motility, we evaluated the velocity and directionality of the lysosomes in the axons of cells injected with a GFP construct and uninjected cells in the same dish. Movement of the lysosomes was tracked using the acidotrophic probe LysoTracker Red DND‐99. Our data show that the direction, velocity and number of moving lysosomes in both the anterograde and retrograde directions are similar in the cells that have been injected as in uninjected cells (Figure 1B,C).

**Table 1:** Summary of 14 different axonally transported cargoes that have been tested in this study and their nature of movements in SCG neurite

Category	Name of cargo candidate	Brief functional description	Nature of movement in SCG neurites	Reference
Signalling	Mitogen‐activated protein kinase kinase 1 (MAP2K1)	An essential component of the MAP kinase signal transduction pathway. Regulate diverse cellular programmes including embryogenesis, proliferation, differentiation and apoptosis	Diffuse	53
	Mitogen‐activated protein kinase kinase 5 (MAP2K5)		Diffuse	
	Bcl‐2‐interacting mediator of cell death (BIM)	A member of the BH3‐only proapoptotic Bcl‐2 family. Induced in cerebellar granule neurons (CGNs) undergoing apoptosis	Diffuse	54
	c‐Jun NH2‐terminal kinase I (JNKI)	Required for embryonic morphogenesis and this signalling pathway contributes to the regulation of cell proliferation and apoptosis	Diffuse	55
	c‐Jun NH_2_‐terminal kinase II (JNKII)		Diffuse	
	c‐Jun NH_2_‐terminal kinase III (JNKIII)		Diffuse	
	Microtubule‐associated protein 1A/1B‐light chain 3 (LC3)	A mammalian homologue of yeast Atg8 (Aut7/Apg8) and serves as a marker protein for autophagosomes	Diffuse	56
Neurodegeneration	Nicotinamide mononucleotide adenylyltransferase 2 (NMNAT2)	A labile axon survival factor whose constant replenishment by anterograde axonal transport is a limiting factor for axon survival	Punctate	57
	Superior cervical ganglia‐10 (SCG10)	A neuron‐specific stathmin protein with a potent microtubule destabilizing factor and is enriched in the growth cones of the developing neurons	Punctate	58
Diseases associated	Amyloid precursor protein (APP)	Takes a central position in Alzheimer's disease (AD) pathogenesis: APP processing generates the β‐amyloid (Aβ) peptides, which are deposited as the amyloid plaques in brains of AD individuals	Punctate	59
	Beta‐secretase 1 (BACE1)	A membrane‐bound enzyme that cleaves full‐length APP at the β‐secretase cleavage site	Punctate	60
	Brain‐derived neurotrophic factor (BDNF)	A type of neurotrophins that are important regulators of neural survival, development, function, and plasticity	Punctate	61
	Superoxide dismutase 1 (SOD1)	Act as antioxidant enzymes whose main role is to intercept and inactivate reactive oxygen species	Diffuse	62
Organelle	Mitochondria	An organelle found in large numbers in most cells, in which the biochemical processes of respiration and energy production occur	Punctate	

SCG, superior cervical ganglions.

Of the 14 cargo candidates that we tested 6 showed a punctate structure that moved bidirectionally within the axons so movement along the axon could be represented by kymographs. Interestingly, all six cargoes whose movements can be tracked easily are involved either in neurodegenerative diseases or neurodegeneration. For example, both NMNAT2 (Nicotinamide Nucleotide Adenylyltransferase 2) and SCG10 (superior cervical ganglia‐10) are involved in the Wallerian degeneration pathway. NMNAT2, which shares the same NAD‐synthesizing enzyme activity with WLD^S^ (slow Wallerian degeneration protein), is now well established as an essential survival factor that prevents spontaneous degeneration of healthy axons. Its constant replenishment from the cell body into axons by anterograde transport is essential for axon survival and its specific depletion is sufficient to induce Wallerian degeneration.^15^

Surprisingly, we found that most of the signalling molecules showed a diffuse signal throughout the axons, so their movements could not be easily tracked. These diffuse signals may have occurred because the majority of the fluorescently labelled molecules were unattached and could diffuse freely through the axon. These unattached cargoes lead to high background noise that makes it particularly difficult to track any individual microtubule‐dependent moving vesicles.^23^

### 2.2 KIF1A has a high percentage of co‐migration with BACE1

Next, we wanted to find the cargoes that co‐migrate with KIF1A. Toward this, we microinjected dissociated SCG cultures with low levels of KIF1A‐GFP/Cargo candidates‐RFP (or mCherry) and simultaneously visualized their transport in axons 6‐8 hours after microinjection where the orientation of the axons in relation to the cell body was unambiguous (schematic in Figure 1A). This is the time when these fluorescently labelled proteins are just beginning to be expressed and the fluorescent protein signals are at relatively low levels. This could avoid the problem of artefacts, which are produced by high levels of expression. To demonstrate this pattern, we measured the average fluorescent intensity of neurites at three different time points after microinjection (6, 24 and 48 h) and have presented images captured at these time points in Figure S1B, Supporting Information. We found that there was an increase in fluorescent intensity with time after microinjection. At 6 hours, the average level of fluorescent intensity was relatively low compared to its levels at 24 or 48 hours (Figure S1C). In addition, to ensure that the signals from the GFP and RFP channels are comparable, we generated line fluorescent intensity profiles along the axon co‐expressing NMNAT2‐GFP and NMNAT2‐mCherry. The profiles generated from the GFP and RFP channels are quantitatively similar in terms of fluorescent peak locations (Figure S2).

**Figure 2:**
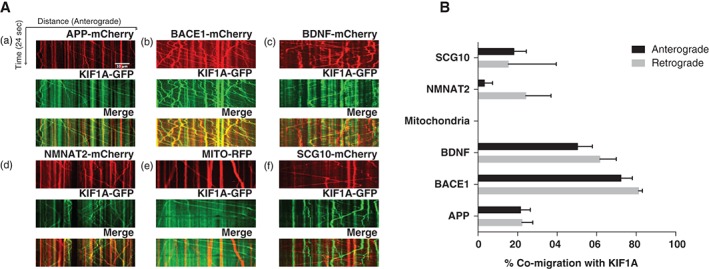
(A) Co‐migration with KIF1A‐GFP was analyzed using kymographs (time‐distance graphs) obtained from live imaging of axons from dissociated superior cervical ganglions (SCG) neurons. Representative kymographs from axons co‐labelled with KIF1A‐GFP and (a) amyloid precursor protein (APP)‐mCherry (b) beta‐secretase 1 (BACE1)‐mCherry (c) BDNF‐mCherry (d) NMNAT2‐mCherry (e) mitochondria‐RFP (f) SCG10‐mCherry. (B) Quantification of co‐migration with KIF1A‐GFP. The quantification shown for each construct represents the percentage of moving vesicles that co‐migrate with KIF1A‐GFP. [amyloid precursor protein (APP): n = 18 neurites, beta‐secretase 1 (BACE1): n = 22 neurites, BDNF: n = 21 neurites, MITO: n = 21 neurites, nicotinamide nucleotide adenylyltransferase 2 (NMNAT2): n = 10 neurites, SCG10: n = 14 neurites). Error bars indicate standard error of the mean (SEM)].

To analyse the extent of co‐migration between KIF1A‐GFP and cargo candidates, kymographs were generated for each channel (GFP and RFP/mCherry) and for their overlays (Figure 2A). To quantify the extent of co‐migration, only particles that moved during at least part of the time‐lapse recording were taken into account. The percentage of co‐migration in the axon was then analyzed by comparing pairs of kymographs generated for each fluorescently tagged cargo. We found that KIF1A had a different percentage of co‐migration with different cargo candidates (Figure 2B): It had the highest percentage of co‐migration with BACE1 but a relatively low percentage of co‐migration with both NMNAT2 and mitochondria. BDNF, APP and SCG10 all showed intermediate levels of co‐migration with KIF1A, with BDNF being most strongly associated of these.

### 2.3 KIF1A is functionally required for the transport of BACE1‐mCherry

The demonstration of a high percentage of co‐migration between KIF1A‐GFP and BACE1‐mCherry raised the question of whether KIF1A is functionally required for transporting BACE1 or whether other motor proteins can substitute when KIF1A activity is removed.

To address this question, we first asked whether the transport of BACE1 vesicles could be disrupted by the expression of a mutant kinesin construct. We expressed a KIF1A‐GFP mutant with a point mutation (Thr‐312 to Met) that impairs the motor domain activity (KIF1A‐T312M‐GFP) in dissociated SCG neurons. This point mutation occurs in the α‐helix 5 in KIF1A that is close to the centre of the surface for binding microtubules.^24^ It impairs the motor activity by interrupting the interactions between the microtubules and kinesin. We observed that most of the KIF1A‐T312M particles were localized in the cell body, and the GFP signal was more restricted to the proximal sections of the axons (Figure S3A). At distances greater than approximately 8 µm from the cell body, a significant decrease in GFP fluorescence was observed in neurites expressing KIF1A‐T312M‐GFP compared with those expressing wild‐type KIF1A‐GFP (Figure S3B). Consistent with previously published data,^14^ the number of KIF1A‐T312M‐GFP moving particles was also significantly reduced in both directions compared with wild‐type KIF1A‐EGFP (Figure S3C).

**Figure 3:**
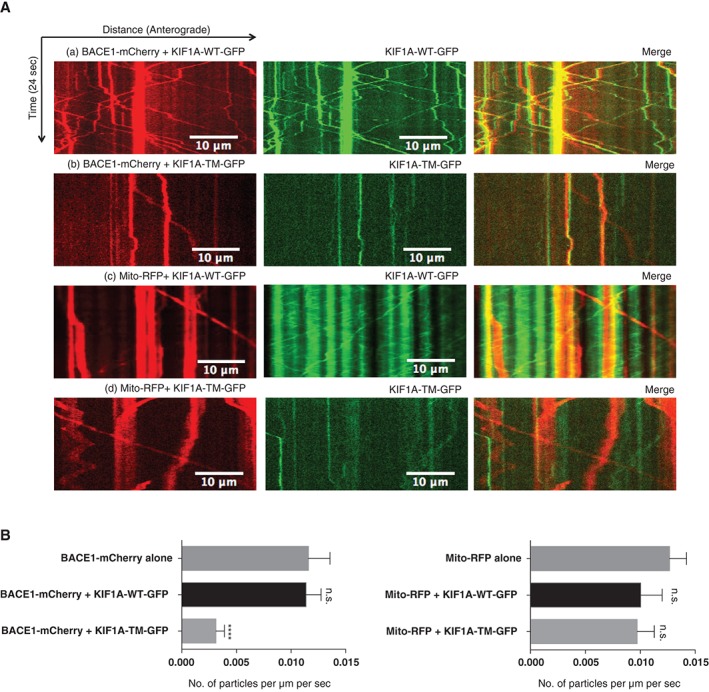
(A) KIF1A‐T312M‐GFP reduces the transport of beta‐secretase 1 (BACE1) vesicles significantly in axons of dissociated superior cervical ganglions (SCG) neurons. Representative kymographs comparing transport of BACE1‐mCherry between (a) KIF1A‐WT‐GFP and (b) KIF1A‐T312M‐GFP expressing neurons. Representative kymographs comparing transport of mito‐RFP between (c) KIF1A‐WT‐GFP and (d) KIF1A‐T312M‐GFP expressing neurons. (B) Quantification of the number of moving BACE1‐mCherry particles (alone: n = 18, with KIF1A‐WT‐GFP: n = 17, with KIF1A‐T312M‐GFP: n = 23, ****P ≤ .0001, t‐test) (bottom left panel). Quantification of the number of moving mitochondria‐RFP particles (alone: n = 10, with KIF1A‐WT‐GFP: n = 28, with KIF1A‐T312M‐GFP: n = 32) (bottom right). Error bars indicate standard error of the mean (SEM).

When KIF1A‐T312M‐GFP was expressed with BACE1‐mCherry, there was a significant reduction in the number of BACE1‐mCherry moving particles in both the anterograde and retrograde directions. We believe that this mutant acts by exerting a dominant negative action on endogenous KIF1A by competing with endogenous KIF1A to bind to BACE1 (Figure 3A,B). However, the expression of KIF1A‐TM‐GFP had no effect on the axonal transport of mitochondria (Figure 3A,B), which was previously found to be a cargo of KIF1B^25^ and KIF5 26, 27, 28, 29 and have 0% of co‐migration with KIF1A (Figure 2B).

**Figure 4:**
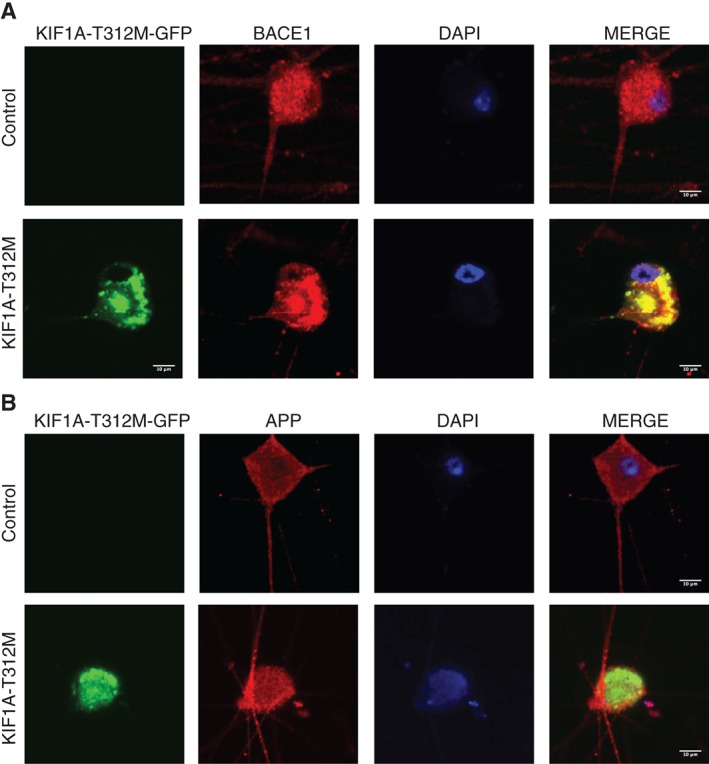
KIF1A‐T312M‐GFP impairs axonal transport of endogenous beta‐secretase 1 (BACE1) but not amyloid precursor protein (APP). KIF1A‐T312M‐GFP expressing neurons or control were immunostained for (A) BACE1 or (B) APP. Representative cells are shown with merge (n = 5, scale bar = 10 µm).

Further, to test whether endogenous BACE1 distribution is affected; we monitored the axonal distribution of BACE1 in neurons expressing KIF1A‐T312M using immunohistochemistry. Compared to the control, there was a shift of BACE1 protein from neurites to cell bodies (Figure 4A), suggesting that the transport of endogenous BACE1 was impaired. However, the expression of mutant KIF1A protein did not appear to affect the localisation of endogenous APP, which had a relatively low percentage of co‐migration with KIF1A (Figure 2B). APP protein was still observed in both the cell body and axonal compartment (Figure 4B).

**Figure 5:**
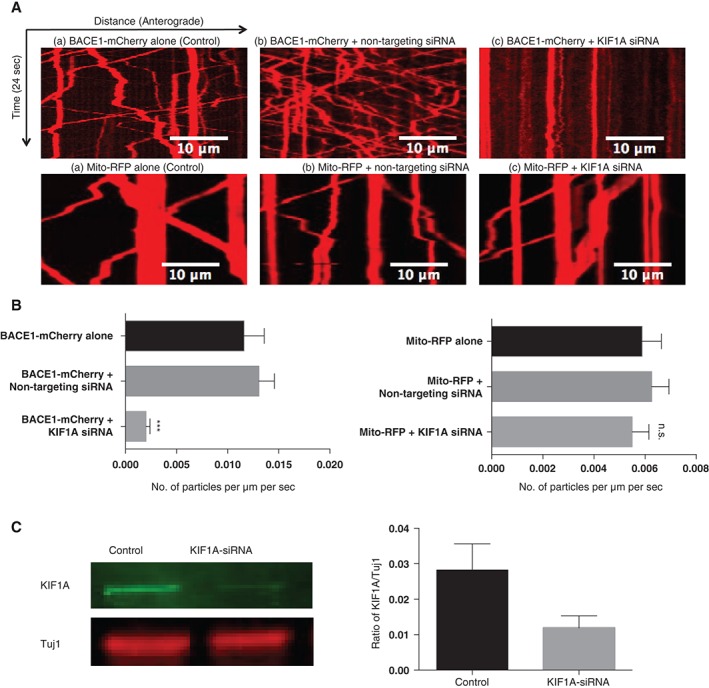
(A) Knockdown of KIF1A inhibits beta‐secretase 1 (BACE1) but not mitochondria movement in axons of dissociated superior cervical ganglions (SCG) neurons. Representative kymographs comparing transport of BACE1‐mCherry between (a) control and (b) non‐targeting siRNA and (c) KIF1A siRNA neurons (upper panel). Representative kymographs comparing transport of mito‐RFP between (a) control and (b) non‐targeting siRNA and (c) KIF1A siRNA neurons (bottom panel). (B) Quantification of the number of moving BACE1‐mCherry particles (control: n = 18, non‐targeting siRNA: n = 11, KIF1A siRNA: n = 11, P < .001, t‐test) (left panel). Quantification of the number of moving mitochondria‐RFP particles (control: n = 12, non‐targeting siRNA: n = 10, KIF1A siRNA: n = 10) (right panel). Error bars indicate standard error of the mean (SEM). (C) Representative immunoblots performed on cell lysates to show knockdown of KIF1A. Quantification of the knockdown of KIF1A is expressed as a ratio of KIF1A to Tuj1 expression. (n = 3). Error bars indicate standard error of the mean (SEM).

Second, we asked whether the depletion of endogenous KIF1A could affect the axonal transport of BACE1 vesicles. We imaged the axonal transport of BACE1‐mCherry in axons co‐expressing siRNA against KIF1A. We observed a significant reduction in BACE1‐mCherry moving particles 48 hours after microinjection compared to cells either without siRNA or with non‐targeting (control) siRNA (Figure 5A,B). To further show the specificity of our siRNA, we also looked at the axonal transport of mitochondria in neurons expressing KIF1A siRNA. We found that the axonal transport of mitochondria is not affected by the non‐targeting siRNA (control) or knocking down of KIF1A protein (Figure 5A,B). These findings suggest that KIF1A activity is functionally required for the transport of BACE1‐mCherry.

### 2.4 BACE1, NMNAT2 and mitochondria are transported in distinct carriers

We previously reported that NMNAT2 and mitochondria move independently of one another.^30^ Interestingly, our data showed that both NMNAT2 and mitochondria also have a very low percentage of co‐migration with KIF1A‐GFP (Figure 2B). This prompted us to ask whether BACE1 is transported by a different carrier from those carrying either NMNAT2 or mitochondria as this may suggest the existence of several independently moving groups of cargoes. We examined the percentage co‐migration of BACE1‐mCherry with NMNAT2‐GFP and also the percentage co‐migration of BACE1‐mCherry with Mitochondria‐RFP using live imaging of each pair of cargoes in the same axon.

**Figure 6:**
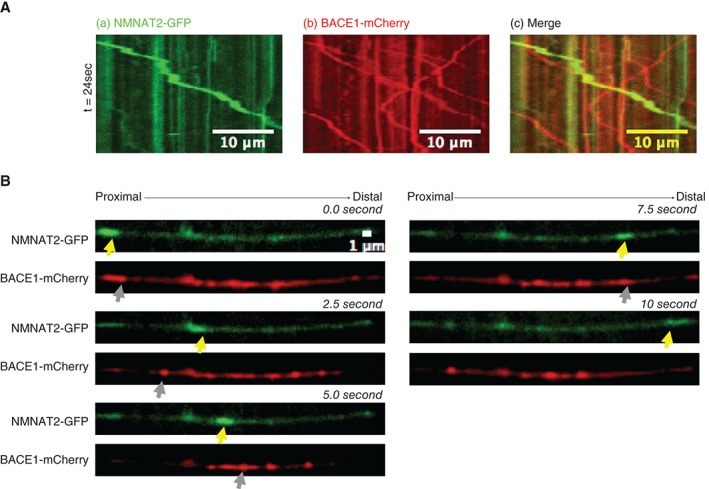
Two‐colour microscopy shows that beta‐secretase 1 (BACE1)‐mCherry and NMNAT2‐GFP are transported in different carriers in co‐injected superior cervical ganglions (SCG) axons. (A) Representative kymographs of (a) NMNAT2‐GFP, (b) BACE1‐mCherry and (c) Overlay of (a) and (b). As shown in the kymographs, the localization of mobile BACE1/NMNAT2 was mostly non‐overlapping. (B) Five frames from the time‐lapse images are shown, with each channel (GFP and RFP) displayed separately. Yellow arrows mark positions of a vesicle containing only NMNAT2‐GFP. Grey arrows mark positions of a vesicle containing only BACE1‐mCherry.

**Figure 7:**
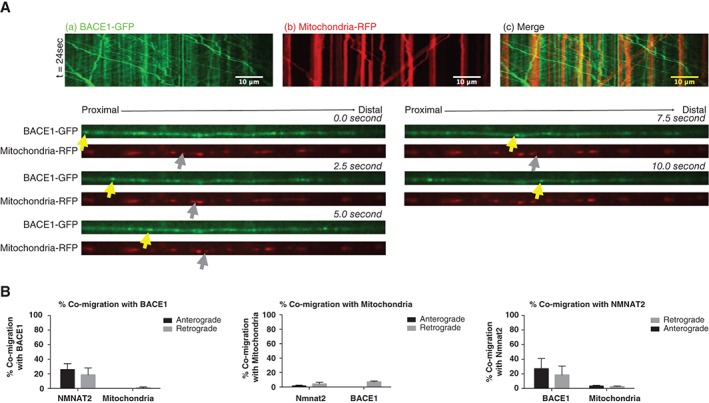
Two‐colour microscopy shows that beta‐secretase 1 (BACE1)‐GFP and mitochondria‐RFP are transported in different carriers in co‐injected SCG axons. (A) Representative kymographs of (a) BACE1‐GFP, (b) mitochondria‐RFP and (c) Overlay of (a) and (b). As shown in the kymographs, the localization of mobile BACE1/NMNAT2 was mostly non‐overlapping. (B) Five frames from the time‐lapse images are shown, with each channel (GFP and RFP) displayed separately. Yellow arrows mark positions of a vesicle containing only BACE1‐GFP. Grey arrows mark positions of a vesicle containing only mitochondria‐RFP. (C) Quantification of co‐migration of (a) BACE1 with NMNAT2 or mitochondria (b) NMNAT2 with BACE1 or mitochondria (c) Mitochondria with BACE1 or NMNAT2. Error bars indicate standard error of the mean (SEM).

In SCGs neurons co‐injected with BACE1‐mCherry and NMNAT2‐GFP (Figure 6A), the localisation of mobile BACE1 and NMNAT2 was mostly non‐overlapping. Consecutive images from a 2.5‐second time‐lapse series are shown in Figure 6B with yellow arrowheads in the GFP channel (Figure 6B) marking an anterogradely moving NMNAT2‐GFP vesicle not containing BACE1‐mCherry. Grey arrowheads in the RFP channel (Figure 6B) mark an anterogradely moving BACE1‐mCherry vesicle not containing NMNAT2‐GFP. Thus, these particular vesicles moving BACE1 and NMNAT2 are transported by distinct carriers and the quantification (Figure 7B) shows this is typical of the large majority of such vesicles. Similar results were found when BACE1‐GFP and mito‐RFP were co‐injected (Figure 7A). Yellow arrowheads in the GFP channel (Figure 7A) mark an anterogradely moving BACE1‐GFP vesicle not containing RFP and grey arrowheads in the RFP channel (Figure 7A) mark an anterogradely moving mitochondria not containing GFP. Thus, BACE1 and mitochondria are also moving independently from one another.

**Figure 8:**
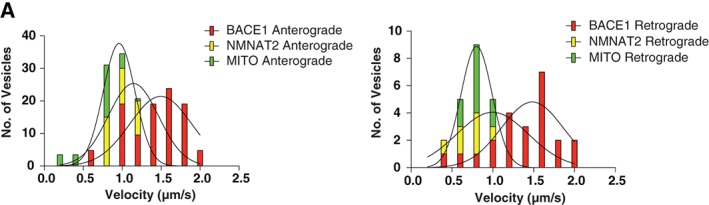
Beta‐secretase 1 (BACE1), nicotinamide nucleotide adenylyltransferase 2 (NMNAT2) and mitochondria vesicles have subtle differences in both anterograde and retrograde transport kinetics. (A) Overlay of the velocity frequency histograms describe the velocity distribution of mobile BACE1 (red bars, n = 24, 905 vesicles), NMNAT2 (yellow bars, n = 21, 408 vesicles) and mitochondria (green bars, n = 29, 443 vesicles).

We also compared the transport kinetics of mobile BACE1‐mCherry/NMNAT2‐mCherry/Mitochondria‐RFP vesicles using a plugin developed by our group in the software imageJ.^31^ Subtle differences were recorded in their average and maximum velocity in both the anterograde and retrograde directions (Figure 8A), especially for moving mitochondria which was the slowest of these cargoes in both anterograde and retrograde direction (summarized in Table 2). Taken together, these findings suggest that BACE1, NMNAT2 and mitochondria are transported in distinct carriers and that KIF1A is a selective kinesin responsible for moving one group of independently moving cargoes in axons.

**Table 2:** Summary table for the axonal transport kinetics of moving fluorescently labelled BACE1, NMNAT2 and mitochondria vesicles

	BACE1‐mCherry	Mitochondria‐RFP	NMNAT2‐mCherry
Morphology	Vesicular (tubular has been observed)	Mainly tubular	Vesicular
Direction	Both anterograde and retrograde	Mainly anterograde	Mainly anterograde
Average velocity (µm/s)	1.40 ± 0.07 (anterograde)	0.93 ± 0.05 (anterograde)	1.21 ± 0.07 (anterograde)
	1.31 ± 0.10 (retrograde)	0.90 ± 0.06 (retrograde)	1.01 ± 0.08 (retrograde)
Maximal velocity (µm/s)	2.45 ± 0.14	1.63 ± 0.06	1.87 ± 0.08

BACE1, beta‐secretase 1; NMNAT2, nicotinamide nucleotide adenylyltransferase 2

## 3 Discussion

### 3.1 BACE1, NMNAT2 and Mitochondria are markers for non‐overlapping components of axonal transport

The live imaging technique was introduced in the late 1980s to overcome the limitations of using whole‐nerve radiolabelling in studying axonal transport. In this approach, DNA constructs encoding transport cargoes fused with fluorescent protein are delivered to neuronal cell bodies, and the movement of the fluorescently labelled cargoes observed directly by time‐lapse imaging. This method achieves a much higher temporal and spatial resolution than the traditional radiolabelling approach. Most importantly, individually moving cargoes can also be tracked.^32^ In this study, we showed that KIF1A is the primary anterograde motor protein mediating the axonal transport of BACE1. To the best of our knowledge, this is the first study to identify the kinesin responsible for moving BACE1 using live cell imaging, a result further supported by the dependence of BACE1 transport on levels of functional KIF1A.

Advances in fluorescence microscopy allow us to understand the distribution and dynamics of different types of axonally transported cargoes in living cells. We can now follow and record the movement of a fluorescently labelled cargo within axons both in vitro and in vivo in real time. 33, 34 For example, the generation of transgenic mice (MitoS mice) that expresses cyan fluorescent protein targeted to mitochondria allows us to visualise and follow the mitochondria movement in individual axons over extended periods of time.^34^ Although mitochondria are useful for the live imaging of axonal transport, they are not representative of most axonal transport cargoes, as their movement may be influenced as much by local energy demands as by transport capacity. Moreover, only a minority of mitochondria move in axons. To address this limitation, we generated a new transgenic mouse to study the axonal transport of NMNAT2, which is an essential survival factor for the maintenance of healthy axons. The transport of NMNAT2 can be assayed in the central nervous system explants and in peripheral nerves. 35, 36 Our data showed that BACE1 also moves independently of both NMNAT2 and Mitochondria. Hence, BACE1 appears to be a useful marker for studying a third, non‐overlapping component of axonal transport. A useful future research direction would be to cross the transgenic mice expressing fluorescently tagged BACE1^37^ with both MitoS and NMNAT2 transgenic models to simultaneously report on multiple axonal transport motors. We could then follow the axonal transport of three independently moving cargoes using time‐lapse imaging. In this way, we would be able to obtain a more comprehensive view of how axonal transport is affected in aging^36^ or in models of neurodegenerative diseases.^38^

### 3.2 Retrograde transport of KIF1A and BACE1

In this study, we show that KIF1A has a high percentage of co‐migration with BACE1 in both the anterograde and retrograde directions. As all of the axonal microtubules are orientated uniformly, with the plus ends (polymerising ends) facing the axon terminals, the observation in this and other studies 13, 14 that the plus‐end driven kinesin KIF1A is capable of moving retrogradely is rather surprising. It has been proposed that cargoes move bidirectionally due to a tug‐of‐war with oppositely directed motor proteins (ie, dynein complex) attached to the same cargo. 39, 40 However, the results that both knocking down KIF1A and expressing the T312M mutant (probably a dominant negative) diminished BACE1 motility in both the anterograde and retrograde directions challenges this model. It may be that without anterograde transport there is less BACE1 available for retrograde transport but this observation also fits with the previously suggested “co‐dependence” model^39^ and with previous studies in which inhibition of *Drosophila* Unc‐104 (KIF1A) disrupts axonal transport of dense core vesicles in both directions.^41^ Instead of competing mechanically with dynein motors, we suggest that KIF1A itself could act as a cargo for the dynein complex. Indeed, a previous study found that KIF1A is a component of the cytoplasmic dynein complex.^42^ We present the first evidence of the bidirectional co‐migration of KIF1A and BACE1. Bidirectional transport of cargo in axons offers distinct biological advantages. For example, it may help to overcome obstacles and explore a large region of cellular space before delivering the cargo to the most appropriate site.^43^

### 3.3 Redundancy among kinesins

As a relatively small number of kinesins have to move a much larger number of transport cargoes, it is clear that each kinesin has multiple roles. Indeed studies in knockout animals have previously implicated KIF1A in the movement of synaptic vesicle precursors.^44^ However, it remains unclear how much redundancy exists between different kinesins in the function of moving an individual transport cargo. Mutation of a single kinesin in humans or mice is in many cases sufficient to cause axonal growth or degeneration phenotypes, indicating that not all roles of the respective kinesin can be compensated for by others. 45, 46, 47 This includes a possible role for KIF5A in the axonal transport of mitochondria, one of the cargoes we find shows minimal co‐migration with KIF1A.^48^ However, there are also indications of some functional redundancy in the movement for example of neurofilaments^49^ and late endosomes.^50^ Thus, it appears likely that the degree of functional redundancy differs on a cargo‐by‐cargo basis.

### 3.4 Role of KIF1A in separating APP and BACE1 trafficking

Despite intensive investigation, the location where wild‐type APP and BACE1 meet and the site of the β‐cleavage are still not completely understood. In our study in SCG neurons, we found that KIF1A has a high percentage of co‐migration with BACE1 (about 70% in the anterograde direction and about 80% in the retrograde direction) but a relatively lower percentage of co‐migration with APP (about 20% in both the anterograde and retrograde direction). Thus, in this neuron subtype at least, overexpressed BACE1 and APP are transported primarily by different kinesins. Moreover, we found that most moving APP and BACE1 vesicles are non‐overlapping (Figure S4). We used dual‐colour, live‐cell imaging of primary SCG neurons to visualise the co‐migration of APP with BACE1. The co‐migration of APP‐GFP and BACE1‐mCherry was then quantified using kymographs; only moving particles were counted. We found that the majority (>50%) of the APP particles were moving separately from the BACE1 particles in the anterograde direction. However, it should be noted that the APP construct used in this study was tagged at the carboxy‐terminal; therefore, this method does not distinguish between the transport of full‐length APP and fragments generated by cleavage that contain the carboxy‐terminal region (CTFs). Similarly, the effect of the overexpression of BACE1 on APP transport is not properly understood. Thus, the question of where APP and BACE1 meet is difficult to resolve using live imaging alone.

Our results do raise the interesting prospect that changes in KIF1A could influence the axonal transport of BACE1, and through this influence the intracellular site and rate of processing of APP. Interestingly, an increase in BACE1 was paradoxically found to lower the production of Aβ,^51^ leading to the suggestion that shifting processing to the cell body, as for example by reducing KIF1A activity (Fig 4A), could be beneficial. In contrast, lowering expression of KLC leads to more Aβ generation, potentially by increasing the dwell time of APP processing products in the axon.^52^ Thus, we suggest that altering the different components of axonal transport that we describe, may have different effects on Alzheimer's disease pathogenesis.

## 4 MATERIALS AND METHODS

### 4.1 Animals

C57BL/6JOlaHsd mice were obtained from Harlan UK (Bicester, UK). All animal work was carried out in accordance with the Animals (Scientific Procedures) Act, 1986, under Project License 80/2254.

### 4.2 Cell cultures

#### 4.2.1 SCG dissection

SCGs were dissected from 0 to days old C57BL/6 (wild‐type) mouse pups. Primary neuronal cell culture medium was prepared from DMEM (glucose: 4500 mg/L, sodium pyruvate: 110 mg/L; Sigma) to which 2 mM glutamine (Invitrogen), 100 ng/mL 7S NGF (Invitrogen), 1% penicillin/streptomycin (Invitrogen), 10% foetal bovine serum (Sigma) were added. 4 μM aphidicolin (Calbiochem) was also used to reduce proliferation and viability of small numbers of non‐neuronal cells. Cultures were used after 5‐7 days.

#### 4.2.2 Dissociated SCG cultures

Dissected SCG ganglia were incubated in 0.025% trypsin (Sigma) in PBS (without CaCl_2_ and MgCl_2_) at 37°C for 30 min. This was followed by incubation in 0.2% collagenase type II (Gibco) in PBS at 37°C for a further 30 min. Ganglia were then gently triturated using a pipette. After a 2‐hour pre‐plating step to remove non‐neuronal cells, 5000‐10,000 dissociated neurons in 40 μL of primary neuronal cell culture medium were plated in a 1 cm^2^ poly‐l‐lysine and laminin‐coated area in the center of 3.5 cm ibidi μ‐dishes (Thistle Scientific) for microinjection experiments. A further 1 mL of primary neuronal cell culture medium was added after 2 hours of incubation. Medium was changed the next day and every 2‐3 days after that.

### 4.3 Plasmids construct and siRNA reagents

Expression vectors encoding EGFP‐tagged SCG10 and BDNF were generated by amplification of the complete coding region of each gene using reverse transcriptase PCR (RT‐PCR) (see below) from 1 µg total RNA from wild‐type mouse brain. Products were cloned into pEGFP‐N1 (BD Biosciences Clontech) to generate SCG10‐EGFP and BDNF‐EGFP expression vector. Sequencing (Cogenics) was performed to confirm the absence of PCR errors. SCG10‐mCherry and BDNF‐mCherry were created by replacing the EGFP coding sequence with the mCherry coding sequence from pmCherry‐NI (Clontech). siRNA sequences directed against mouse KIF1A mRNA (ON‐TARGET*plus* Mouse Kif1a SMART pools L‐046928‐01‐0005) were purchased from Dharmacon (Thermo Scientific). Each pool consisted of four individual siRNAs. The target sequences (sense strand) of each siRNA were as follows: (1) 5′‐ACACAUAUGUCAACGGCAA‐3′, (2) 5′‐UGUCCAAAAUAUUGCGGUA‐3′, (3) 5′‐GAAGGAAGUGCGCGAGCUA‐3′, (4) 5′‐GGAGAUUUACUGUGAGCGA‐3′.

ON‐TARGETplus non‐targeting siRNA pool (D‐001810‐10) purchased from Dharmacon (Thermo Scientific) was used as a negative control in experiments.

The MEK1‐GFP, MEK5‐GFP, JNKI‐GFP, JNKII‐GFP, JNKIII‐GFP, mitochondria‐RFP, NMNAT2‐EGFP, NMNAT2‐mCherry, constructs were kindly provided by Dr Jon Gilley and Dr Stefan Milde. The APP‐EGFP and SOD1‐GFP constructs were kindly provided by Prof. Christopher Miller, BACE1‐YFP construct were kindly provided by Professor Gopal Thinakaran. Both KIF1A‐EGFP and KIF1A‐T312M‐EGFP constructs were kindly provided by Dr Jae‐Ran Lee.

### 4.4 Reverse transcriptase PCR

Primers used for detection of SCG10 and BDNF transcripts in SCG neuron RNA were as follows: SCG10: 5′‐ATGGCTAAAACAGCAATGGCC‐3′ and 5′‐CCAGACAGTTCAACCTGCAG‐3′; BDNF: 5′‐ATGACCATCCTTTTCCTTAC‐3′ and 5′‐CTTCCCCTTTTAATGGTCAG‐3′.

### 4.5 Western blotting

Protein levels of KIF1A were assessed by lysing control and siRNA expressing dissociated cortical cultures in 50 μL of phosphate buffered saline with cOmplete, ethylenediaminetetraacetic acid (EDTA)‐free protease inhibitor cocktail tablets (Sigma‐Aldrich). Protein samples were vortexed and then heated in Laemmli buffer to 100°C for 10 min. A total of 15 µl of each sample were separated on a 12% SDS‐PAGE. After transferring the gel to PVDF membrane, the membrane was probed with a polyclonal anti‐KIF1A antibody (Abcam; 1:1000) and a monoclonal anti‐beta tubulin antibody (Sigma; 1:2000). After three washes with PBST for 10 minutes each, bands were detected with AlexaFluor680‐conjugated anti‐mouse secondary antibody (Molecular Probes, Eugene, Oregon). Membranes were then scanned and quantified using the Odyssey imaging system (LI‐COR Biosciences, Lincoln, North Carolina).

### 4.6 Microinjection of DNA constructs

Microinjection mixes of plasmid DNA were prepared in 0.5× PBS(−) and passed through a Spin‐X filter (Costar). Eppendorf Femtotips were loaded with the microinjection mix and injection was performed using an Eppendorf 5171 transjector and 5246 micromanipulator system on a Zeiss Axiovert 200 microscope. All injections were carried out directly into the nuclei of dissociated SCG neurons. For live cell imaging, a maximum total DNA concentration of 0.05 µg/μL in the injection mix was used. A total of 75 cells were injected per dish and imaging was performed 6‐8 hours after microinjection. As on only the injected cells express fluorescent protein, no other marker of injection is required.

### 4.7 Immunofluorescence and live imaging

For immunostaining, cells were fixed in 4% paraformaldehyde (PFA) in phosphate‐buffered saline (PBS) for 30 min, washed in PBS three times and permeabilized with Triton X‐100 for 5 min. The cells were then blocked with 50% goat serum (Sigma) in PBS for 30 min and probed with primary antibodies diluted in blocking solution for 1 hour at room temperature. APP: Merckmillipore 22C11 at 1:200 dilution; BACE1: Abcam ab2077 at 1:100. Cells were then washed in PBS three times before probing with goat anti‐mouse or anti‐rabbit secondary antibody coupled to Alexa Fluor 568 (1:500). Confocal images were acquired using a Zeiss LSM780 confocal microscope with a 20× objective.

For live cell imaging, cells were transferred (on the day of imaging) into imaging medium in order to improve performance and detectability of fluorescent proteins. Cells were viable and appeared morphologically normal in imaging medium for at least 2 days. Imaging medium consisted of 1.80 mM CaCl_2_ (Sigma), 0.25 μM Fe(NO_3_)_3_ (Sigma), 0.81 mM MgSO_4_ (AnalaR), 5.33 mM KCl (AnalaR), 44.05 mM NaHCO_3_ (Sigma), 110.34 mM NaCl (AnalaR), 0.92 mM NaH_2_PO_4_ (Sigma), 4500 mg/l glucose (AnalaR), 110 mg/L sodium pyruvate(PAA), 2 mM glutamine (Invitrogen), 100 ng/mL 7S NGF (Invitrogen), 1% penicillin/streptomycin (Invitrogen), 4 μM aphidicolin (Calbiochem), 1× MEM amino acids (PAA), 30 mg/L glycine (AnalaR) and 42 mg/L serine (Sigma) in sterile distilled water.

Neuronal lysosomes were visualized by incubation of 100 nM LysoTracker Red DND‐99 (Molecular Probes) in fresh medium for 30 minutes at 37°C. Neurons were washed and then replaced with fresh culture medium before imaging.

Time‐lapse images were acquired using an Andor wide‐field/TIRF imaging system comprising Nikon Ti‐E microscope, Nikon 100×1.49 TIRF objective, Andor laser combiner, Andor TuCam image splitter and 2× Andor iXon EMCCD cameras. Cultures were maintained at 37°C in an OKO labs environment chamber. Time‐lapse images of proximal axon regions were captured for 24 seconds with a 250 ms time‐lapse interval using iQ3 live cell imaging software (Andor). A total of 10‐12 movies from different neurons were captured from each culture dish. The software imageJ was used for generation of kymograph and analysis of particle velocities.

### 4.8 Data analysis

Statistical analyses and graph fitting were performed using graph pad prism 6.0 (GraphPad Software Inc.)

## Supporting information

Editorial Process

**Figure S1**. (A) Schematic of experiments. Neurons were imaged at three different time points (6, 24 and 48 hours) after microinjection. (B) Representative images of axon expressing nicotinamide nucleotide adenylyltransferase 2 (NMNAT2)‐mCherry at 6, 24 and 48 hours after microinjection. (C) Average fluorescence intensity along the axons is measured at 6, 24 and 48 hours after microinjection (n = 3 neurites). Error bars indicate standard error of the mean (SEM).
**Figure S2**. Fluorescent signal from the GFP and RFP channels are comparable. (A) Representative images and (B) lines fluorescent intensity profiles along axon co‐expressing nicotinamide nucleotide adenylyltransferase 2 (NMNAT2)‐GFP and NMNAT2‐mCherry. We show that the profiles generated from the GFP and RFP channels are quantitatively similar in terms of fluorescent peak locations.
**Figure S3**. Movements of KIF1A are impaired by the point mutation (T312M). (A) SCG neurons microinjected with (a) KIF1A‐WT‐GFP (b) KIF1A‐T312M‐GFP. Higher magnification views of the selected areas in (a) and (b) are shown below. (B) Quantification of the relative GFP fluorescence in the axons at increasing distance from the cell body. At distances greater than 8 µm away from the cell body, GFP signal was significantly reduced (**P ≤ .01, t‐test) in cells expressing KIF1A‐T312M‐GFP compared to KIF1A‐WT‐GFP. Error bars indicate standard error of the mean (SEM) (n = 10). (C) Quantification of the number of moving KIF1A particles. The number of moving KIF1A‐T312‐GFP particles (n = 24 neurites from 2 to 3 separate cultures) was significantly reduced when compared to the number moving KIF1A‐WT‐GFP particles (n = 16 neurites from 2 to 3 separate cultures). Error bars indicate SEM.
**Figure S4**. Over half of the moving amyloid precursor protein (APP) and beta‐secretase 1 (BACE1) vesicles are transported in different carrier in the anterograde direction. (A) Representative kymographs from simultaneous APP‐GFP and BACE1‐mCherry imaging. Overlay the two different channels of kymographs. (B) Quantification of co‐migration between APP‐GFP and BACE1‐mCherry. Percentage of APP co‐migrates with BACE1 = 43.32% ± 7.26% (anterograde), 25.07% ± 14.89% (retrograde). Percentage of BACE1 co‐migrates with APP = 43.67% ± 14.37% (anterograde), 52.83% ± 11.75% (retrograde) (n = 6). Error bars indicate standard error of the mean (SEM).
