# Supplementary material for: KIF1A mediates axonal transport of BACE1 and identification of independently moving cargoes in living SCG neurons
Source: Traffic. 2016 Oct 5;17(11):1155–67. doi: 10.1111/tra.12428 (PMC5132087; doi:10.1111/tra.12428)
Supplement: Supplementary file 2 — Figure S1. (A) Schematic of experiments. Neurons were imaged at three different time points (6, 24 and 48 hours) after microinjection. (B) Representative images of axon expressing nicotinamide nucleotide adenylyltransferase 2 (NMNAT2)‐mCherry at 6, 24 and 48 hours after microinjection. (C) Average fluorescence intensity along the axons is measured at 6, 24 and 48 hours after microinjection (n = 3 neurites). Error bars indicate standard error of the mean (SEM). Figure S2. Fluorescent signal from the GFP and RFP channels are comparable. (A) Representative images and (B) lines fluorescent intensity profiles along axon co‐expressing nicotinamide nucleotide adenylyltransferase 2 (NMNAT2)‐GFP and NMNAT2‐mCherry. We show that the profiles generated from the GFP and RFP channels are quantitatively similar in terms of fluorescent peak locations. Figure S3. Movements of KIF1A are impaired by the point mutation (T312M). (A) SCG neurons microinjected with (a) KIF1A‐WT‐GFP (b) KIF1A‐T312M‐GFP. Higher magnification views of the selected areas in (a) and (b) are shown below. (B) Quantification of the relative GFP fluorescence in the axons at increasing distance from the cell body. At distances greater than 8 µm away from the cell body, GFP signal was significantly reduced (**P ≤ .01, t‐test) in cells expressing KIF1A‐T312M‐GFP compared to KIF1A‐WT‐GFP. Error bars indicate standard error of the mean (SEM) (n = 10). (C) Quantification of the number of moving KIF1A particles. The number of moving KIF1A‐T312‐GFP particles (n = 24 neurites from 2 to 3 separate cultures) was significantly reduced when compared to the number moving KIF1A‐WT‐GFP particles (n = 16 neurites from 2 to 3 separate cultures). Error bars indicate SEM. Figure S4. Over half of the moving amyloid precursor protein (APP) and beta‐secretase 1 (BACE1) vesicles are transported in different carrier in the anterograde direction. (A) Representative kymographs from simultaneous APP‐GFP and BACE1‐mCherry imaging. Overlay th [file TRA-17-1155-s001.docx]

**KIF1A mediates axonal transport of BACE1 and identification of independently moving cargoes in living SCG neurons**

Christy O.Y. Hung^1^ and Michael P. Coleman^1,2,*^

^1^ Signalling Programme, Babraham Institute, Cambridge CB22 3AT, UK

^2^ John van Geest Centre for Brain Repair, University of Cambridge, Robinson Way, Cambridge CB2 0PY, UK

Correspondence: Professor Michael Coleman

[mc469@cam.ac.uk](mailto:mc469@cam.ac.uk)

John van Geest Centre for Brain Repair,

University of Cambridge,

Robinson Way,

Cambridge

CB2 0PY

United Kingdom

Supplemental Information

Supplemental Figure 1. (A) Schematic of experiments. Neurons were imaged at three different time points (6 hours, 24 hours and 48 hours) after microinjection. (B) Representative images of axon expressing NMNAT2-mCherry at 6 hours, 24 hours and 48 hours after microinjection. (C) Average fluorescence intensity along the axons is measured at 6 hours, 24 hours and 48 hours after microinjection (n=3 neurites). Error bars indicate SEM.

Supplemental Figure 2. Fluorescent signal from the GFP and RFP channels are comparable. (A) Representative images and (B) lines fluorescent intensity profiles along axon co-expressing NMNAT2-GFP and NMNAT2-mCherry. We show that the profiles generated from the GFP and RFP channels are quantitatively similar in terms of fluorescent peak locations.

Supplemental Figure 3. Movements of KIF1A are impaired by the point mutation (T312M). (A) SCG neurons microinjected with (a) KIF1A-WT-GFP (b) KIF1A-T312M-GFP. Higher magnification views of the selected areas in (a) and (b) are shown below. (B) Quantification of the relative GFP fluorescence in the axons at increasing distance from the cell body. At distances greater than 8µm away from the cell body, GFP signal was significantly reduced (** p ≤ 0.01, t test) in cells expressing KIF1A-T312M-GFP compared to KIF1A-WT-GFP. Error bars indicate SEM. (n=10) (C) Quantification of the number of moving KIF1A particles. The number of moving KIF1A-T312-GFP particles (n=24 neurites from 2-3 separate cultures) was significantly reduced when compared to the number moving KIF1A-WT-GFP particles (n=16 neurites from 2-3 separate cultures). Error bars indicate SEM.

Supplemental Figure 4. Over half of the moving APP and BACE1 vesicles are transported in different carrier in the anterograde direction. (A) Representative kymographs from simultaneous APP:GFP and BACE-1:mCherry imaging. Overlay the two different channels of kymographs. (B) Quantification of co-migration between APP:GFP and BACE1:mCherry. Percentage of APP co-migrates with BACE1 = 43.32% ± 7.26% (Anterograde), 25.07% ± 14.89% (Retrograde). Percentage of BACE1 co-migrates with APP = 43.67% ± 14.37% (Anterograde), 52.83% ± 11.75% (Retrograde) (n=6) (Error bars indicate SEM.)
