# Supplementary material for: KIF1A mediates axonal transport of BACE1 and identification of independently moving cargoes in living SCG neurons
Source: Traffic. 2016 Oct 5;17(11):1155–67. doi: 10.1111/tra.12428 (PMC5132087; doi:10.1111/tra.12428)
Supplement: Supplementary file 1 — Editorial Process [file TRA-17-1155-s002.docx]

| Title: | **KIF1A mediates axonal transport of BACE1 and identification of independently moving cargoes in living SCG neurons** |
| --- | --- |
| Authors: | Christy O.Y. Hung and Michael P. Coleman |
| Article Type: | Original Research |
|  |  |
| Monitoring Editor | Trina Schroer |
| Date Submitted | 22 March, 2016 |
| Date for Decision 1 | 25 April, 2016 |
| Date Resubmitted | 21 June, 2016 |
| Date for Decision 2 | 15 July, 2016 |
| Date Resubmitted | 27 July, 2016 |
| Accepted | 29 July 2016 |

**Decision and Reviews**

Dear Dr. Coleman,

Thank you for submitting your manuscript “KIF1A mediates axonal transport of BACE1 and identification of independently moving cargoes in living SCG neurons” for consideration for publication in Traffic. I asked two colleagues who are experts in the field to review the paper. The comments of referee 1 are appended below. Referee 2 is not responding to reminders to complete his/her review. Given the delay in the referee’s response, I have opted to make a decision based on the comments we have in hand. I will forward the comments of referee 2 if they arrive in the next week or so.

I share the enthusiasm of referee 1 for the work presented in this paper. The referee raises a concern about the relative expression levels in these experiments that you will need to address. In addition, the referee has made a few suggestions to improve the presentation and correct the citations.

Although I cannot accept your manuscript for publication at this point, I believe that you will be able to address the referees concerns and I look forward to receiving your revised manuscript. To expedite handling when you resubmit please be sure to include a response outlining how you have addressed each of the referees’ concerns.

Sincerely,

Trina A. Schroer, Ph.D.
Co-Editor
________________________________________________________
Referee's Comments to the Authors

Referee: 1

Comments to the Author
The manuscript by Hung and Coleman examines the transport of various cargoes in cultured mammalian neurons by the kinesin motor KIF1A. Previous work has implicated this motor in the transport of a variety of axonal cargoes and the authors continue this line of work. The data is generally clear and well-done. The work is appropriate for Traffic. I have just a few comments that should be addressed to improve the manuscript.

Figures 6 and 7 examine the co-transport of several cargoes. Given the authors interest in whether APP and BACE are transported together, it seems important to do the co-transfection and analysis of these proteins.

The authors state that their microinjection of plasmid DNA allows “expression levels at a minimum” and “two proteins … to be expressed at a consistent ratio”. The former does not appear to be true since the images show quite a lot of fluorescence signal in the cells. Are these images enhanced? Can the authors provide images of cells expressing low, medium or high levels of expression so that the images shown can be put in context? For the latter statement, quantification must be provided in which the difference fluorescence outputs of the fluorescent proteins is taken into account. A GFP-tagged protein may be expressed at low levels but show similar fluorescence levels because GFP is brighter than mCherry. The expression of a fluorescently-tagged protein typically depends more on the proteins stability than the amount of input plasmid.

I don’t understand the x-axes in Figure 3B (and similar graphs later in the paper). The legend says that the number of moving particles was counted (~10 per condition). I have no idea what “average count per frame per 160 um” means. Average count of what? 160 um where? And I am surprised that the transport of mito-RFP when co-expressed with KIF1A-TM-GFP is not significantly different than the mito-RFP alone (Figure 3). The data seem to be clearly different. Why is the KIF1A-TM having an effect on mito-RFP transport if KIF1A does not transport mitochondria?

Some of the citations are questionable, leading one to question the authors’ knowledge of the subject area. For example, there are many references describing cargoes carried by KIF1A (top of right column, p. 2). There are many excellent reviews on kinesins other than the quoted 15-year old review (bottom of left column, p. 2).

Figure 2 could be moved to the supplement

_____________________________________________________________________________**Author Rebuttal**

Dear Sir/Madam,

We thank the reviewers for their helpful comments. The manuscript has been thoroughly revised, with inclusion of new data in Figs 1(B&C), 2, 3, 5 and 6, and two Suppl. Figs, together with text revisions and detailed answers to reviewers’ points.

In brief, Reviewer 1 considered the work appropriate for Traffic but asked about the expression levels of our DNA plasmids, in particular our suggestion that we minimise overexpression issues by imaging 6-8h after microinjecting them. We now show in Fig 2 that this is indeed the earliest stage when fluorescently-tagged proteins are visible enough to quantify axonal transport and that the levels continue to increase substantially after this time. We also show that our ability to detect red and green fluorescence is similar (new Fig 3).

Reviewer 2 questioned how this advances understanding of neurodegenerative pathology. This is an important question but quite different from the gap in published studies that we address here: the assignment of specific cargoes to specific kinesins by asking how much they comigrate in live imaging analysis and whether knockdown of a specific kinesin reduces their movement. Thus, with the editor’s agreement (email discussion of 4th and 5th May), we have revised the manuscript in accordance with this aim and discussed the implications for Alzheimer’s disease in the Discussion. We prefer at this stage to report a conclusive result about the kinesin-cargo relationship rather than attempting to resolve this controversial, wider question too, which will be a large study in its own right. To the best of our knowledge, this is the first study to identify KIF1A as the primary kinesin responsible for the axonal transport of BACE1, the first to report co-migration of these proteins by live imaging and the first report of KIF1A siRNA influencing BACE1 anterograde and retrograde transport. Future studies could build on our findings to explore the implications for Alzheimer’s disease.

We are very grateful to the reviewers for their comments and we believe this has substantially strengthened the manuscript. We hope you will now find it suitable for publication in Traffic.

Sincerely,

Professor Michael Coleman,
John van Geest Centre for Brain Repair Cambridge CB2 0PY
UK

Comments from Reviewer 1:
(1) Figures 6 and 7 examine the co-transport of several cargoes. Given the authors interest in whether APP and BACE are transported together, it seems important to do the co-transfection and analysis of these proteins.

Response: Thank you for this direction. We agree that this is an important area to address and we have indeed done this experiment (now included as Supplemental Figure 2). However, considering the controversial history of this problem (outlined in the last paragraph of the manuscript), and the practical complications of addressing it (see below), a conclusive statement would require significantly more supporting data and diverge from the intended purpose of our manuscript. Thus, while we discuss this at the end of the Discussion, we wish to stop short of making this a main conclusion.

Our finding on this question is that in our system the majority of APP particles were moving separately from the BACE1 particles in the anterograde direction, which of course agrees with some studies but not others. However, it should be noted that the APP construct used in this study was tagged at the carboxy-terminal; therefore, this method does not distinguish between the transport of full-length APP and fragments generated by cleavage that contain the carboxy-terminal region (CTFs). Likewise, the effect of the overexpression of BACE1 on APP transport is not properly understood. As the question of where APP and BACE1 meet has been a controversial problem and would need further evidence (in addition to further live imaging studies) to resolve convincingly.

(2) The authors state that their microinjection of plasmid DNA allows “expression levels at a minimum” The former does not appear to be true since the images show quite a lot of fluorescence signal in the cells. Are these images enhanced? Can the authors provide images of cells expressing low, medium or high levels of expression so that the images shown can be put in context?

Response: This is a good suggestion, which we have addressed by imaging neurites at three different timepoints after microinjection (6, 24 and 48 hrs) (new Figure 2). There is a clear increase in fluorescent intensity with time after microinjection, corresponding to low, medium and high expression levels as suggested. The 6-8h timepoint chosen for our experiments is a good match for when fluorescently labelled proteins are just beginning to be expressed, thereby minimising overexpression artefacts.

(3) and “two proteins ... to be expressed at a consistent ratio”. For the latter statement, quantification must be provided in which the difference fluorescence outputs of the fluorescent proteins is taken into account. A GFP-tagged protein may be expressed at low levels but show similar fluorescence levels because GFP is brighter than mCherry. The expression of a fluorescently- tagged protein typically depends more on the proteins stability than the amount of input plasmid.

Response: The Reviewer raises a very valid point about the correlation between fluorescent intensity and plasmid concentration. Without having an independent measure of expression level on a western blot (which we cannot do because only a few dozen neurons in each dish are microinjected) it is difficult to be sure expression is very similar so we have now removed this statement from our manuscript. However, to confirm that our ability to detect both red and green signals is similar, we generated line fluorescent intensity profiles along axons co-expressing both NMNAT2-GFP and NMNAT2-mCherry. The profiles generated from the GFP and RFP channels are quantitatively similar in terms of fluorescent peak locations and in the ability to detect even the weaker peaks.

(4) I don’t understand the x-axes in Figure 3B (and similar graphs later in the paper). The legend says that the number of moving particles was counted (~10 per condition). I have no idea what “average count per frame per 160 um” means. Average count of what? 160 um where?

Response: Thank you for this point. In the revision, we have changed the x-axes labels to ‘number of moving particles per um per second’, along with a corresponding change in absolute numbering. We hope this wording is more self explanatory.

(5) And I am surprised that the transport of mito-RFP when co-expressed with KIF1A-TM-GFP is not significantly different than the mito-RFP alone (Figure 3). The data seem to be clearly different. Why is the KIF1A-TM having an effect on mito-RFP transport if KIF1A does not transport mitochondria?

Response: We agree that there was a strong trend in the original dataset, even if not beyond the threshold for significance. In order to collect a larger, and thus more representative sample, we performed three further independent experiments (involving the analysis of an additional 18 videos). The combined data (now in Fig 5B) again show the expression of KIF1A-T312M-GFP has no significant effects on mitochondria movement, and now there is only a very weak trend relative to wild-type control. As this is also suggested by the KIF1A siRNA experiment (Fig 7) we are confident that KIF1A is not a major carrier for mitochondria.

(6) Some of the citations are questionable, leading one to question the authors’ knowledge of the subject area. For example, there are many references describing cargoes carried by KIF1A (top of right column, p. 2). There are many excellent reviews on kinesins other than the quoted 15-year old review (bottom of left column, p. 2).

Response: Thank you for this comment. We have updated the reference in the revised manuscript.

(7) Figure 2 could be moved to the supplement

Response: Thanks for this suggestion. We have moved Figure 2 to the supplementary section in the revised manuscript.

Comments from Reviewer 2:

On the point about advancing understanding of neurodegeneration, please see cover letter. We agree this is an important question so we have added a supplemental figure and some discussion. However, we think it is important to stop short of changing the focus of the paper, which is addressing the gap in knowledge of axonal transport using methods (live imaging of comigration and siRNA) that have a lot to add to this field but have so far been underused. Thus, with the editor’s agreement we have revised the manuscript according to that purpose.

(1) Introduction: the authors should specify what is BACE1 in the introduction instead of in the conclusion/discussion. Also, more emphasis is needed to provide rationale for choosing BACE-1 as a model cargo for KIF1A. What is the biomedical significance of BACE-1? How those 14 cargoes candidates were chosen?

Response: Thank you for this valuable comment. In the revised introduction, we specify what BACE1 is and provide a rationale for choosing BACE-1 as a model cargo for KIF1A. We also explain that we chose the particular 14 cargoes tested in our study because we wanted to include cargoes with interesting functions from a wide range of categories, ranging from signalling molecules (e.g. MAP2K1, MAP2K5, BIM, JKNI, JNKII, JNKIII and LC3) to proteins that are involved in neurodegenerative disorders (e.g. APP, BACE1, BDNF and SOD1). The focus on signalling relates to a strong interest in signalling pathways at our host institute, providing a convenient source of some fluorescently tagged plasmids and wider interests in the results.

(2) Results #1: no figure number was mentioned

Response: The figure number is now included.

(3) (a) Results #2: Authors used co-migration or comigration. They should chose one spelling.

Response: The spelling “Co-migration” is used throughout the revised manuscript.

(3) (b) The authors mentioned NMNAT2 in this section but gave the definition in paragraph #4 of the results.

Response: We now give the definition of NMNAT2 in paragraph #1 of the Results section.

(4) Figure 1C: the authors only specified the results for BACE1, NMNAT2 and mitochondria, what about for APP, BDNF and SCG10?

Response: The revised manuscript now comments on all six cargoes (the former Fig 1C is now Fig 4C).

(5) Results #4: Figures 5,6 and 7 are composed with different panels A, B and C; the authors only mentioned one sentence for each figure. The authors should describe each panel more in detail explaining arrows, kymographs, and different graphs, to help the reader to understand their results and conclusions.

Response: The last two paragraphs of the Results section in our revised manuscript now describes these figures (now Figs 8, 9 and 10) in much more detail.

(6) Methods: Which microscope/objective were used for live imaging? Explain the protocol of live imaging: one image every X seconds?
What is the DNA sequence of the KIF1A siRNA and scrambled?

Response: Apologies for this oversight. A much more detailed description of methodology including these points is provided in the revised manuscript. Dharmacon does not release the sequences of the non-targeting siRNA pool (which we have renamed from ‘scrambled’) but the full product information for ordering is now given in the manuscript.

(7) Results #1: How were the microinjected neurons identified post-injection? The authors have used double labeling with GFP and RFP plasmids but the microinjection dye was not mentioned.

Response: Microinjection dye is not required because only injected cells can express fluorescent protein. (Injection is the only way cells can obtain the GFP- and RFP-tagged expression plasmids.) Therefore, all of the cells that expressed a fluorescent signal were injected; un-injected cells cannot give any signals. We have clarified this point in the Methods.

(8) What tests were used to determine whether the microinjected neurons were healthy? Is visual evaluation of cellular health by phase contrast sufficient? Alternatively, the authors can inject an empty GFP vector in neurons and quantify the velocity and directionality of lysotracker red vesicles in these injected and uninjected neurons. This will determine if the microinjection is affecting the vesicle motility in neurons. This is especially important, as the neurons will be only analyzed 6-8 hours after injection.

Response: We agree this is an important control and thank the reviewer for this suggestion. As suggested, we have quantified the average velocity and number of moving lysosomes in both the anterograde and retrograde directions (Fig 1B,C) to determine whether microinjection affects vesicle motility through cell damage or other mechanisms. We evaluated the velocity and directionality of the lysosomes in the axons of the injected cells compared to uninjected cells in the same dish. Movement of the lysosomes was tracked using the acidotrophic probe LysoTracker Red DND-99. Our data show that the direction, velocity and number of moving lysosomes in both the anterograde and retrograde directions in injected cells are similar to those in uninjected cells.

(9) How do the authors explain a high percentage of KIF1A and BACE1 comigration in the retrograde direction? Do the authors think that the KIF1A here is a cargo for the retrograde dynein motor or is undergoing a tug-of-war with dynein?

Response: This is a very interesting question, which we now expand on in the revised Discussion (second paragraph). In this study, we show that KIF1A has a high percentage of co-migration with BACE1 in both the anterograde and retrograde directions. As all of the axonal microtubules are orientated uniformly, with the plus ends (polymerising ends) facing the axon terminals, the observation in this and other studies that plus-end driven kinesin-like KIF1A is capable of moving retrogradely is rather surprising. It has been proposed that cargoes move bidirecitonally due to a tug- of-war with oppositely directed motor proteins (i.e. dynein complex) attached to the same cargo. However, the result that knocking down KIF1A diminished BACE1 motility in both the anterograde and retrograde directions challenges this model. This finding is in line with previous studies of Drosophila in which inhibition of Unc-104 (KIF1A) disrupts axonal transport of dense core vesicles in both directions. Instead of competing mechanically with dynein motors, we believe that KIF1A itself may act as a cargo for the dynein complex. Indeed, a previous study found that KIF1A is a component of the cytoplasmic dynein complex. We present the first evidence of the bidirectional co-migration of KIF1A and BACE1. Bidirectional transport of cargo in axons offers distinct biological advantages. For example, it may help to avoid obstacles and explore a large region of cellular space.

(10) The authors refer to KIF1A as a monomeric motor, but this has been definitively disputed by the work of Hammond and Verhey.

Response: Thanks for pointing this out. We have added this to the second paragraph of the Introduction and cited the paper in the revised manuscript.

(11) What is the authors’ explanation for a reduction in bidirectional transport of BACE-1 upon KIF1A-T312M expression?

Response: When KIF1A-T312M-GFP was expressed with BACE1-mCherry, there was a significant reduction in the number of BACE1-mCherry moving particles in both the anterograde and retrograde directions. We believe that this mutant acts by exerting a dominant negative action on endogenous KIF1A by competing with endogenous KIF1A to bind to BACE1. The reduction in retrograde transport of BACE1-mCherry is likely to have a similar explanation to that after KIF1A siRNA (see point 9) so the mutant is mentioned in the same Discussion paragraph.

(12) Have the authors looked at the endogenous distribution of BACE-1 and APP in control and KIF1A mutant neurons? This experiment will help determine the effect of impaired BACE-1 transport on APP processing.

Response: We agree this is an important control for the effects of overexpression, which, although we did minimise it as demonstrated in the new Fig 2, is unavoidable for the live imaging experiments. Thus, to test whether endogenous BACE1 distribution is affected; we monitored the axonal distribution of BACE1 in neurons expressing KIF1A-T312M by immunohistochemistry. We observed a redistribution of BACE1 out of axons into cell bodies (Fig 6) suggesting that transport of endogenous BACE1 was impaired. However, the expression of mutant KIF1A protein did not appear to affect the localisation of endogenous APP, consistent with its relatively low percentage of co-migration with KIF1A (Fig 4). APP protein was still observed in both the cell body and axonal compartment. Impairment of BACE1 axonal transport may indeed have potential to alter APP processing, a point we now mention in the Discussion, but we believe testing whether it actually does so lies outside the scope of this paper, focussed on linking specific cargoes to specific kinesins.

(15) (a) Fig.1 panel B: the font for figure labels should be larger.

Response: This has now been amended (the Figure is moved to Fig 4).

(15) (b) What was the longest duration of imaging for kymographs? Why only a 24 second time frame was chosen for analysis?

Response: Time-lapse images were captured for 24 s with a 250 ms time-lapse interval. We imaged transport for 24 seconds because the imaging chamber is not connected to any CO2 supply. Ten to twelve videos are normally captured per culture dish (a point now added to the Methods) and it generally takes a few minutes to locate the cell body of an individual neuron. A 24-second time frame was chosen to reduce the total amount of imaging time without compromising the amount of information we could retrieve and to keep any variations that occurred due to a reduction in carbon dioxide levels as low as possible. Of course by imaging multiple neurons in each dish we do apply a much longer total imaging time but with the added advantage of averaging out variation between one neuron and another.

(15) (c) Some kymographs (apart from mito) show large vesicles (> 3uM), how do the authors explain the heterogeneity in vesicle size for the same marker?

Response: This is a very good point. It is not unusual to find heterogeneity in the sizes of axonally transported vesicles even for the same cargo. This was previously seen, for example, for BACE1 (Figures 4&5 (Buggia-Prévot, Fernandez et al. 2014); Figure 4 (Das, Wang et al. 2016)), NMNAT2 (Supplementary video (Gilley and Coleman 2010)) and APP (Video 1 (Fu and Holzbaur 2013)). To some extent in any still image this reflects vesicles that are temporarily passing one another and appear as a single vesicle, which in a movie can be seen to be separate. However, it also appears to reflect heterogeneity among transported vesicles. In addition, it has been reported that the longitudinal size of vesicles decreases as the vesicles move through an axon (Nakata, Terada et al. 1998), suggesting that some of the large vesicles might divide into smaller vesicles while being transported to the distal side of axons.

(16) Fig. 2 panel B: 32-40 uM bars should be significantly different.

Response: Thank you for spotting this. On reanalysing the data we did indeed find a significant difference in intensity at the distance of 32-40μM (P value = 0.0076). This is now shown in the figure (now moved to supplementary Figures at the suggestion of Reviewer 1).

(17) Fig. 2 panel C: what is the unit of measure for X-axis?

Response: This was a measure of the density of moving particles, but in the revision, we have changed the x-axis labelling in this and other figures to ‘number of moving particles per um per second’ which should be more self-explanatory.

(18) Fig. 2 panel A: Why the cell body is so brightly labeled?

Response: Protein synthesis within the cell body, before transport of some of the protein into axons, is likely to be the reason why cell bodies are brightly labelled. This is not unusual for proteins that undergo axonal transport.

(19) Fig.3 panel A: The KIF1A and merged channels should be shown.

Response: This has been done in the revised manuscript (the figure is now Fig 5).

(20) Fig. 3 panel B: the difference between mito -RFP alone and Mito-RFP + KIF1A-TM-GFP may be significant.

Response: We agree that there was a strong trend in the original dataset, even if not beyond the threshold for significance. In order to collect a larger, and thus more representative sample, we performed three further independent experiments (involving the analysis of an additional 18 videos). The combined data (now in Fig 5B) again show the expression of KIF1A-T312M-GFP has no significant effects on mitochondria movement, and now there is only a very weak trend relative to wild-type control. As this is also suggested by the KIF1A siRNA experiment (Fig 7) we are confident that KIF1A is not a major carrier for mitochondria.

(21) Fig. 4 panel A: Which region of axon was imaged to generate the kymographs? Was the distribution of BACE1 altered before and after siKIF1A?

Response: The proximal region of axons was imaged to generate the kymographs and this is now stated in the Methods. Our live imaging studies suggest that a significant proportion of BACE1 accumulates in the cell body after loss of KIF1A. We observed a significant reduction in BACE1 moving particles 48 hours after microinjection compared to cells either without siRNA or with non-targeted siRNA (Now Figure 6A,B).

(22) Fig. 4 panel C: Why was the signal intensity compared between Tuj1 and KIF1A, when the blot shows tubulin and KIF1A? The authors should show Tuj1 blot, if it was used for quantification.

Response: Tuj1 is the clone number given to the monoclonal antibody that reacts specifically with class III beta-Tubulin (55 kDa) (i.e. Tuj1 represents the antibody used to show the expression level of tubulin). Following your suggestion, we have changed the label of the western blot from tubulin to Tuj1 in the revised manuscript (now Fig 7).

(23) Fig.5 panel B: increase contrast for the motility tracks or show gray scale. Also, mention the scale bar on these panels.

Response: This has been done. We thank the reviewer for this suggestion to improve the figure (now Fig 8)

(24) Fig.7 panels (a), (b), (c) and (d): show the frequency distribution with paired bar graphs. The current representation is complicated to obtain quantitative information from.

Response: The layout of this Figure (now Fig 10) has been improved as suggested.

Reference
Buggia-Prévot, V., C. G. Fernandez, S. Riordan, K. S. Vetrivel, J. Roseman, J. Waters, V. P. Bindokas, R. Vassar and G. Thinakaran (2014). "Axonal BACE1 dynamics and targeting in hippocampal neurons: a role for Rab11 GTPase." Molecular neurodegeneration 9(1): 1.
Das, U., L. Wang, A. Ganguly, J. M. Saikia, S. L. Wagner, E. H. Koo and S. Roy (2016). "Visualizing APP and BACE-1 approximation in neurons yields insight into the amyloidogenic pathway." Nature neuroscience 19(1): 55-64.
Fu, M.-m. and E. L. Holzbaur (2013). "JIP1 regulates the directionality of APP axonal transport by coordinating kinesin and dynein motors." The Journal of cell biology 202(3): 495-508.
Gilley, J. and M. P. Coleman (2010). "Endogenous Nmnat2 is an essential survival factor for maintenance of healthy axons." PLoS Biol 8(1): e1000300.
Nakata, T., S. Terada and N. Hirokawa (1998). "Visualization of the dynamics of synaptic vesicle and plasma membrane proteins in living axons." The Journal of cell biology 140(3): 659-674.

_____________________________________________________________________________**Decision and Reviews**

Dear Dr. Coleman,

Thank you for submitting your revised manuscript “KIF1A mediates axonal transport of BACE1 and identification of independently moving cargoes in living SCG neurons” to Traffic. I asked the referees to read the revised paper. The verbatim comments of referee 1 are appended below. I share the view of the referees that you have addressed the concerns raised previously. Referee 1 makes several recommends to clarify and correct the presentation that you will need to address. Referee 2 recommends accepting your paper in confidential comments.

Although I cannot accept your manuscript for publication at this point, I believe that you will be able to address the referee’s concerns and I look forward to receiving your revised manuscript in the very near future. To expedite handling when you resubmit please be sure to include a response outlining how you have addressed each of the referees’ concerns.

Sincerely,

Trina A. Schroer, Ph.D.
Co-Editor
________________________________________________________
Referee's Comments to the Authors


Referee: 1

Comments to the Author
The authors have done an excellent and thorough job addressing the concerns of the reviewers. Overall, this is a nice study, focused and clear in its questions and experiments, with quantifiable results. This work provides an analysis of cargo transport by KIF1A in mammalian neurons and the first analysis of BACE-1 transport. It is thus suitable for Traffic. I have just a couple of minor suggestions to improve clarity and presentation.

Bottom left, page 2: Kif1A does NOT belong to the KIF1A/Unc104 family. It belongs to the kinesin-3 family (Lawrence et al JCB 2004).

Top left, page 3: The sentence "microinjected a low level of the fluorescently labelled construct into the nuclei..." is confusing. The methods indicates that plasmid DNA was microinjected. Was the plasmid fluorescently labelled before microinjection or is the protein that is expressed off the plasmid fluorescently labelled?

same sentence: "visualised its transport" What is "its"? The microinjected plasmid?

Some of the figures lack information about n values (# cells, # images, etc)

Figure 2 can be moved to the supplement since it just shows a control. The time line suggests that the same cell was imaged at different time points. Is this true? Were all of these images taken under the same imaging conditions (length of image acquisition, brightness, etc)? Is part C from one axon or an average across many axons? Please provide standard deviations for the graph in part c in order to show the variability in fluorescence intensities.

Figure 3 ccan be moved to the supplement since it shows a control (not data).

The white labels for the images in Figures 4, 5, S1 are difficult to read. Please use black text outside of the image.

The time courses in Figures 8 and 9 are difficult to analyze with the different colors next to each other. Please rearrange so the two colors can be directly compared at each time point.

Figure 10 is confusing. Both parts A and B are not needed since they are the same data.


Referee: 2

Comments to the Author
(There are no comments.)

_____________________________________________________________________________**Author Rebuttal**

Re: Traffic Manuscript TRA-16-0498.R1 ‘KIF1A mediates axonal transport of BACE1
and identification of independently moving cargoes in living SCG neurons’.

Dear Sir/Madam,

We thank the reviewers for their helpful comments. The manuscript has been thoroughly revised, with text revisions and detailed answers to reviewers’ points. We believe this has substantially strengthened the manuscript. We hope you will now find it suitable for publication in Traffic.

Sincerely,
Professor Michael Coleman,
John van Geest Centre for Brain Repair
Cambridge CB2 0PY
UK

Comments from Reviewer 1:

(1) Bottom left, page 2: Kif1A does NOT belong to the KIF1A/Unc104 family. It belongs to the kinesin-3 family (Lawrence et al JCB 2004).

Response: Thanks for pointing this out. We corrected the sentence and cited the paper in the revised manuscript.

(2) Top left, page 3: The sentence "microinjected a low level of the fluorescently labelled construct into the nuclei..." is confusing. The methods indicates that plasmid DNA was microinjected. Was the plasmid fluorescently labelled before microinjection or is the protein that is expressed off the plasmid fluorescently labelled?

Response: Thank you for this comment. We changed the sentence to “microinjected a low level of construct encoding fluorescently labelled protein into the nuclei…”

(3) same sentence: "visualised its transport" What is "its"? The microinjected plasmid?

Response: We changed the same sentence to “visualized the transport of fluorescently labelled protein”.

(4) Some of the figures lack information about n values (# cells, # images, etc)

Response: Apologies for this oversight. The n values is now included in Fig 2(B), 4 and S1.

(5) (a) Figure 2 can be moved to the supplement since it just shows a control.

Response: Thanks for this suggestion. We have moved Figure 2 (now Supplemental Fig 1) to the supplementary section in the revised manuscript.

(5) (b) The time line suggests that the same cell was imaged at different time points. Is this true? Were all of these images taken under the same imaging conditions (length of image acquisition, brightness, etc)?

Response: Cells from the same cultural dish was imaged under the same imaging conditions at different time points.

(5) (c) Is part C from one axon or an average across many axons? Please provide standard deviations for the graph in part c in order to show the variability in fluorescence intensities.

Response: Part C is from an average across three axons (n=3). We have now included the SEM in the graph.

(6) Figure 3 ccan be moved to the supplement since it shows a control (not data).

Response: Thanks for this suggestion. We have moved Figure 3 (now Supplemental Fig 2) to the supplementary section in the revised manuscript.

(7) The white labels for the images in Figures 4, 5, S1 are difficult to read. Please use black text outside of the image.

Response: The layout of these three figures (now Fig 2, 3 and S3) has been amended as suggested.

(8) The time courses in Figures 8 and 9 are difficult to analyze with the different colors next to each other. Please rearrange so the two colors can be directly compared at each time point.

Response: The layout of these two figures (now Fig 6 and 7) has been improved as suggested.

(9) Figure 10 is confusing. Both parts A and B are not needed since they are the same data.

Response: Part A of this figure (now Fig 8) has been removed for better clarification.

_____________________________________________________________________________
